# Audio-Based Automatic Giant Panda Behavior Recognition Using Competitive Fusion Learning

**DOI:** 10.3390/s25133878

**Published:** 2025-06-21

**Authors:** Yuancheng Li, Yong Luo, Qijun Zhao, Mingchun Zhang, Yue Yang, Desheng Li

**Affiliations:** 1 State Forestry and Grassland Administration Key Laboratory of Conservation Biology for Rare Animals of the Giant Panda State Park, China Conservation and Research Center for the Giant Panda, Chengdu 611830, China; 2 College of Computer Science, Sichuan University, Chengdu 610065, China; 3 National Key Laboratory of Fundamental Science on Synthetic Vision, Sichuan University, Chengdu 610065, China

**Keywords:** behavior recognition, giant panda, bioacoustics, competitive fusion learning

## Abstract

Automated giant panda (*Ailuropoda melanoleuca*) behavior recognition (GPBR) systems are highly beneficial for efficiently monitoring giant pandas in wildlife conservation missions. While video-based behavior recognition attracts a lot of attention, few studies have focused on audio-based methods. In this paper, we propose the exploitation of the audio data recorded by collar-mounted devices on giant pandas for the purpose of GPBR. We construct a new benchmark audio dataset of giant pandas named abPanda-5 for GPBR, which consists of 18,930 samples from five giant panda individuals with five main behaviors. To fully explore the bioacoustic features, we propose an audio-based method for automatic GPBR using competitive fusion learning. The method improves behavior recognition accuracy and robustness, without additional computational overhead in the inference stage. Experiments performed on the abPanda-5 dataset demonstrate the feasibility and effectiveness of our proposed method.

## 1. Introduction

The giant panda (*Ailuropoda melanoleuca*) is a unique and endangered species endemic to China. With the advancement of captive breeding techniques, the reintroduction of captive pandas into the wild has become a growing focus for biologists. To enhance the survival of giant pandas in the wild, a well-designed selection mechanism is essential [1]. The various behaviors of pandas observed during a monitoring period serve as one of the key criteria for selection [2].

Traditionally, the identification of panda behaviors primarily relies on experts manually analyzing and labeling video or audio data. However, this approach is very time-consuming and requires professional training to have the ability to judge behavioral types with high accuracy, and it is prone to subjective bias [3]. Recently, advancements in deep learning have introduced new solutions that can effectively assist researchers. For example, Swarup et al. [4] proposed a method to automatically identify the behavior of captive giant pandas using images, achieving 90% accuracy for behavior classification and 84% for facial motion recognition. Liu et al. [5] proposed a model called PandaFormer to use identify the behavior of wild giant pandas based on videos and obtained 92.25% Top-1 accuracy.

Despite its impressive accuracy, video-based technique requires high quality images of pandas, which are difficult to collect in the wild. In addition, environmental factors such as season, weather, and lighting can seriously degrade image quality, making recognition more difficult [5]. In contrast, using a collar to record audio signals whenever the panda exhibits a certain behavior proves to be a simpler alternative [6]. However, the recorded audio data pose challenges in post-processing, as they often contain significant noise and are less intuitive for behavior labeling compared to video. Using audio-based methods to recognize giant panda behaviors also faces several challenges. (1) There are many environmental noises (such as collar friction, background sound, sounds made by other animals, etc.) that can seriously suppress the acoustic signals related to behavior, making it difficult to extract meaningful features. (2) The sounds produced by giant pandas’ behaviors are complex, containing many basic acoustic events, such as breathing sounds, footstep sounds, chewing sounds, etc. Moreover, the sounds produced by different giant panda individuals, even though these pandas are presenting the same behaviors, have a certain degree of difference. As a consequence, simply applying existing audio recognition methods directly to GPBR tasks probably results in low recognition accuracy and unstable performance when facing new individuals.

In this paper, we propose a novel audio-based GPBR method armed with competitive fusion learning that enables the model to extract richer advanced combinations of features in deep-integrated hidden space, so as to achieve higher accuracy in distinguishing different behaviors and minimize the impact of individual differences.

Overall, we make the following main contributions.

- We construct a giant panda behavior audio dataset called abPanda-5, which includes recordings from five individual pandas and five main behaviors, totaling 18,930 audio samples.
- We use a non-stationary noise reduction algorithm to preprocess the input audio to reduce the interference of noise and propose a novel audio-based GPBR method armed with competitive fusion learning, which uses a dual-branch structure to compete with each other to improve the model’s ability to extract more complex acoustic features without causing additional computational overhead in the inference stage.
- We conduct comprehensive evaluation experiments and analyses on abPanda-5. The results show that our proposed method achieved an average accuracy of 92.98%±2.58% and an average F1-score of 92.99%±2.20%, better than that of counterpart methods. In particular, it is confirmed that our method is also effective on some lightweight models, making it possible to deploy IoT devices to monitor giant pandas in remote areas.

The rest of this paper is organized as follows. Section 2 briefly reviews related work. Section 3 describes the materials and methods used. Experiments and results are presented and discussed in Section 4. Finally, conclusions are drawn in Section 5.

## 2. Related Work

With the continuous advancement of artificial intelligence technologies in the field of computer science, many researchers have applied speech recognition algorithms to the field of animal acoustics [7]. For example, Sun et al. [8] applied convolutional neural networks (CNNs) with data augmentation and transfer learning to classify animal sounds in a tropical rainforest, demonstrating that these techniques can significantly improve accuracy even with small and imbalanced training datasets and are thus feasible for conservation projects. García-Ordás et al. [9] proposed a method using fully convolutional neural networks (FCNs) for multispecies bird sound recognition. While most work has focused on species classification based on animal vocalizations, some researchers attempt to exploit single-species vocalizations. For example, Chen et al. [10] proposed TransformerCNN for domestic pig sound classification and achieved recognition accuracy, AUC, and recall scores of 96.05%, 98.37%, and 90.52%, effectively outperforming traditional models.

Particularly in the study of giant pandas, Zhao et al. [11] proposed a SENet-based model for the automatic recognition of giant panda age and sex from vocalizations, achieving F1-scores of 96.46% for age and 85.85% for sex recognition. Liao et al. [12] proposed a novel deep neural network called 3Fbank-GRU to automatically recognize giant panda vocalizations based on wide spectrum features to accurately label large datasets of giant pandas, achieving over 95% recognition accuracy. Yan et al. [13] proposed an automatic method for predicting giant panda mating success based on acoustic features, demonstrating that using audio-based emotion recognition technology in wildlife conservation is feasible.

However, there is no precedent for using deep learning to recognize giant panda behaviors from sounds. There are some successful experiences that can be learned from studies on other animals, though they are also very rare. For example, Nunes et al. [14] developed a system using wearable sensing and BiLSTM to distinguish chew and bite events in horses. It achieved 94.13% accuracy for chew identification and 88.64% for bite identification. However, their approach demonstrates limitations when processing audio signals generated by complex behaviors and requires substantial computational resources and suffers from slow processing speeds.

In summary, although many acoustic models have been applied in the field of animal acoustics to help researchers achieve good results in animal monitoring and protection, existing methods still cannot achieve excellent results when solving GPBR tasks because they cannot effectively capture more advanced expressions of basic acoustic events in audio signals in deep-integrated hidden space.

## 3. Materials and Methods

### 3.1. Data Collection

This study was conducted with full ethical approval from the committee member of the experimental animal ethics review committee of the China Conservation and Research Center for the Giant Panda (Approval No. CCRCGP2025003). The data used in this paper were acquired at the China Conservation and Research Center for the Giant Panda, State Forestry and Grassland Administration Key Laboratory of Conservation Biology for Rare Animals of the Giant Panda State Park, Sichuan, China.

Considering factors such as battery life, device weight, and storage, we chose Sony ICD-PX333M (Sony Corporation, Tokyo, Japan) devices to record and attach to the collar. The audio data collected was stored as MP3 files with a sampling rate of 44,100 Hz [15] and a compression ratio of 192 kbps. The dataset includes continuous audio from five adult female giant pandas over a period of three days. According to the suggestion of giant panda researchers, we defined the behavior of this study as occurring for more than 30 s. In order to facilitate the follow-up experiments, we adopted mono-audio and cut the audio data into neat 1-min segments. After filtering out a few irrelevant or difficult-to-label segments, each clip was manually labeled as one of five behaviors: eating, resting, moving, nursing, or drinking, following the definitions of giant panda behaviors provided by Kleiman et al. [16]. A total of 18,930 audio samples are obtained, forming the dataset abPanda-5. Detailed information is provided in Table 1.

### 3.2. Overview of the Proposed Method

**Figure 1:**
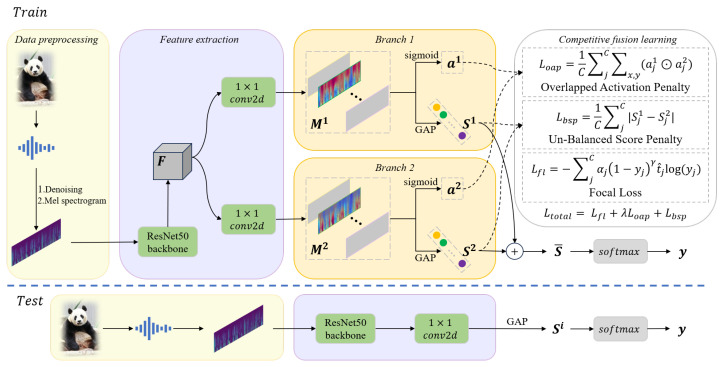
Diagram of the proposed method. The input audio is first preprocessed with denoising and extracting the Mel spectrogram *L*. Subsequently, *L* is fed into the ResNet50 backbone network to extract deep features $F\in R^{h\times w\times d}$. These features are then processed by two branches to obtain class activation maps (CAMs), denoted as $M^{i}\in R^{h\times w\times C}$, where i∈{1,2}. Each $M^{i}$ is passed through a sigmoid function to generate the mask $a^{i}\in R^{h\times w\times C}$, and through global average pooling (GAP) to compute the class scores $S^{i}\in R^{1\times C}$. The two branches are trained with competitive fusion learning constraints imposed by $L_{oap}$, $L_{bsp}$, and $L_{fl}$. Finally, the mean of $S^{i}$ is passed through a softmax function to generate the classification output *y*. During the testing stage, only one branch is required to obtain the recognition result.

Figure 1 shows the flow chart of the proposed method, which begins with the preprocessing of the original audio data, including non-stationary noise reduction and Mel spectrogram conversion, and then extracts deep features through the ResNet50 backbone network. To enable the model to capture more basic acoustic events related to behavior, rather than focusing solely on the most salient acoustic events, we designed a dual-branch neural network with competitive fusion learning, which competed with each other in the training process to learn diverse fine-grained feature expression and advanced feature combinations in different feature sets. The features of shallow learning of neural network represent basic primitives (for example, time–frequency texture), and the shared backbone network reduces the model complexity while maintaining the feature representation ability.

In the training stage, the deep features are sent to two branches to obtain class scores and class activation maps, and the competitive fusion learning method is used for back propagation training. In this process, λ is reduced from 1 thread to 0, which means that the competition gradually weakens, the two branches gradually penetrate and finally converge to the same, and the expression of the feature learned in the competition process will not be forgotten. After the training is completed, only one branch is needed for inference to obtain the recognition result.

### 3.3. Data Preprocessing

#### 3.3.1. Non-Stationary Noise Reduction

Audio data often contain significant amounts of noise. Therefore, noise suppression was carried out to reduce the impact of background noise. We employ a non-stationary noise reduction algorithm for denoising, which has been shown to have better denoising performance in bioacoustics [17].

This method operates on the spectrogram S(f,t) of the input signal, where *f* represents frequency and *t* represents time. A time-smoothed version of the spectrogram $S_{smooth}\left(f,t\right)$ is computed using an Infinite Impulse Response (IIR) filter applied forward and backward on each frequency channel:

(1) $$S_{smooth}\left(f,t\right)=IIR\left(S(f,t)\right).$$

The noise spectrogram is estimated as N(f,t), representing the background noise in the signal. We enhance the signal as follows:

(2) X(f,t)=S(f,t)·G(f,t),

where the gain function G(f,t) is defined as follows:

(3) $$G\left(f,t\right)=\max\left(1-\frac{N(f,t)}{S_{smooth}\left(f,t\right)},0\right).$$

**Figure 2:**
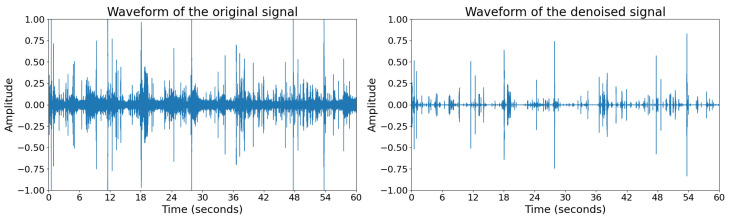
Waveforms of the original signal and denoised signal of an eating audio.

Finally, the denoised time-domain signal $\hat{x}\left(t\right)$ is obtained by performing the inverse Short-Time Fourier Transform (STFT). This method effectively removes non-stationary noise while preserving the essential characteristics of the target signal. Figure 2 shows the waveforms of the original signal and denoised signal.

#### 3.3.2. Mel Spectrogram

To represent the time–frequency characteristics of the audio signal, we extract Mel spectrogram features for each audio sample. Mel spectrogram features are a more compact, robust, and suitable representation that can obtain a high-precision statistical model. First, we applied a pre-emphasis filter to the audio signal $\hat{x}\left(t\right)$ to enhance high-frequency components, compensating for their attenuation, and divided the signal into frames. Subsequently, we applied the Fast Fourier Transform (FFT) to each frame to obtain a power spectrum P(f). Then, the power spectrum is passed through a set of triangular filters mapped to the Mel scale. The center frequency $f_{m}$ of each filter is defined as follows:

(4) $$f_{m}=2595\cdot \log_{10}\left(1+\frac{f}{700}\right).$$

For each filter, the energy is computed to obtain the Mel spectrogram M(m):

(5) $$M\left(m\right)=\sum_{f=f_{m}-1}^{f_{m}+1}P\left(f\right)\cdot H_{m}\left(f\right),$$

where $H_{m}\left(f\right)$ is the frequency response of the *m*-th filter.

**Figure 3:**
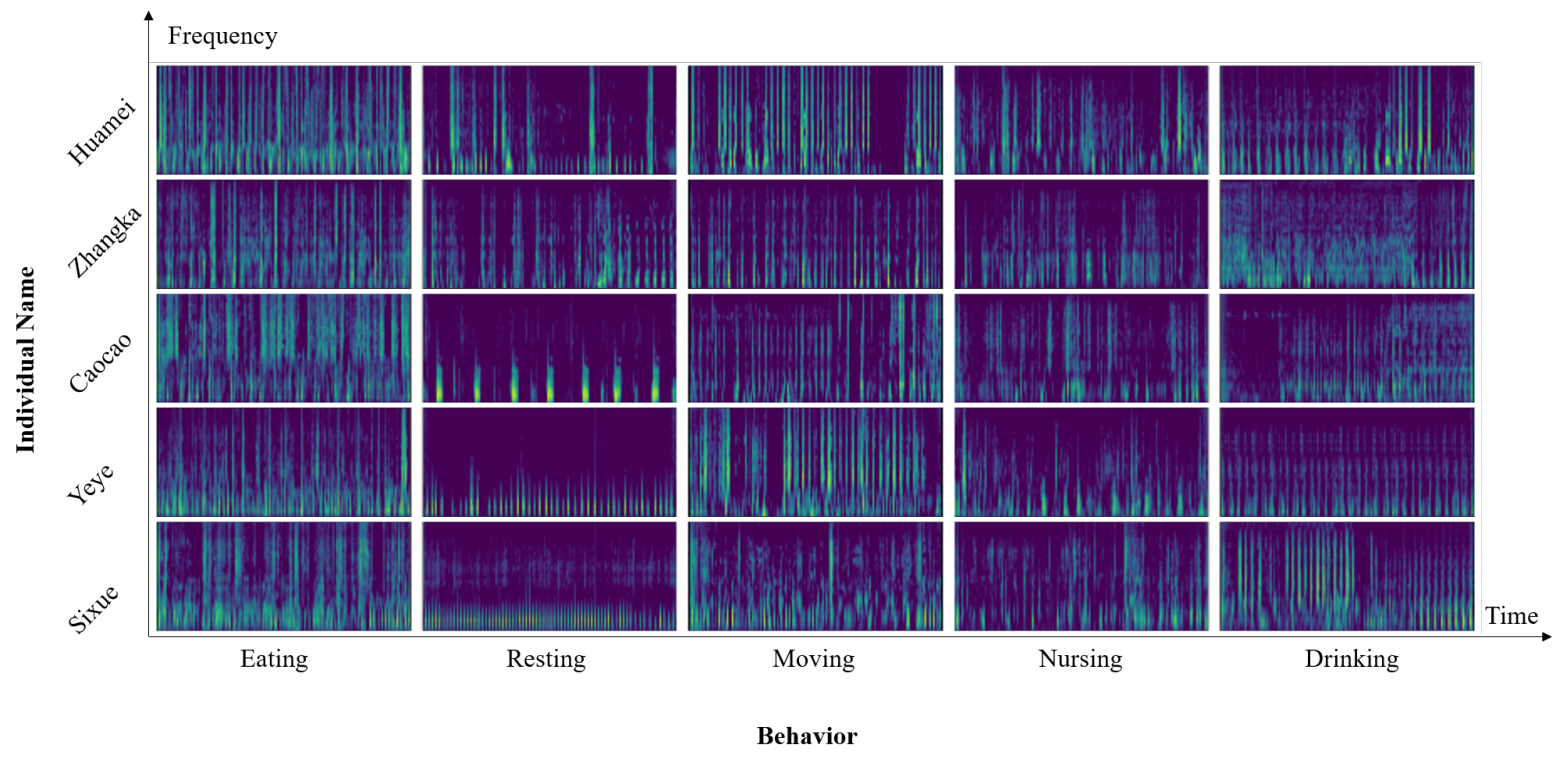
The Mel spectrogram of different individuals’ different behaviors.

The resulting Mel spectrogram features are used for the subsequent classification task. The Mel spectrogram holds significant biological relevance and interpretability and possesses extensive applications in various fields such as sound recognition and audio classification [18]. The Mel spectrograms of selected audio samples are shown in Figure 3.

### 3.4. Competitive Fusion Learning

#### 3.4.1. Baseline Model

In this study, the ResNet50 [19] backbone is employed as the feature extractor. The input Mel spectrogram *L* passes through the ResNet50 backbone network to obtain a tensor $F\in R^{h\times w\times d}$, which can be interpreted as dense h×w grids of *d*-dimensional local features $F\left(x,y\right)\in R^{1\times d}$ at the spatial location (x,y). In the original ResNet50 baseline model, the feature map *F* is first processed by global average pooling (GAP) and then passed through a fully connected (FC) layer to obtain the class scores $S\in R^{1\times C}$, where *C* denotes the number of classes. The baseline process can be expressed as follows:

(6) S=FC(GAP(F)).

#### 3.4.2. Class Activation Maps

Class activation maps (CAMs) [20] are a widely used technique in visual models for visualizing the regions of an image that the neural network focuses on when making classification decisions. Here, we use a 1×1 2D convolution followed by GAP to obtain the class scores *S*, replacing the original approach of applying GAP first and then passing the feature vector through a FC layer to obtain the class scores. The derivation of this process can be represented as follows:

(7) $$\begin{array}{cc} S_{j} & =FC(GAP(F))\\ \; & =\sum_{k=1}^{d}W_{j,k}\frac{1}{h\times w}\sum_{x,y}F_{k}\left(x,y\right)\\ \; & =\frac{1}{h\times w}\sum_{x,y}\sum_{k=1}^{d}W_{j,k}F_{k}\left(x,y\right)\\ \; & =GAP(conv(F)), \end{array}$$

where $W_{j,k}$ represents the weight connecting the *k*-th input channel to the *j*-th output channel in the 1×1 2D convolution layer and is equivalent to the parameters of the FC layer.

The activation map for class *j*, denoted as $M_{j}\left(x,y\right)$, representing the contribution score of the local feature $F\left(x,y\right)\in R^{1\times 1\times d}$ to the class score $S_{j}$, is defined as follows:

(8) $$M_{j}\left(x,y\right)=\sum_{k=1}^{d}W_{j,k}F_{k}\left(x,y\right).$$

#### 3.4.3. Loss Function

For each input audio spectrogram *L*, the two branches are used to obtain the CAMs, $M^{1}$ and $M^{2}$. Then, the sigmoid function is applied to each spatial location (x,y) in *M* to obtain the masks $a^{1}$ and $a^{2}$. The values of these masks are normalized between zero and one, where values closer to one indicate a higher relevance of the corresponding region in L to the target behavior. This process can be described as follows:

(9) $$a\left(x,y\right)=\frac{1}{1+\exp(-M(x,y))}.$$

To encourage the two branches to compete with each other and extract richer class-related features, we minimize the overlap between the high-response regions of $a^{1}$ and $a^{2}$. This forces each branch to focus more on the low-response regions to learn more class-related basic acoustic events. To achieve this, we introduce the Overlapped Activation Penalty (OAP) [21] to penalize the overlapping area of $a^{1}$ and $a^{2}$, defined as follows:

(10) $$L_{oap}=\frac{1}{C}\sum_{j=1}^{C}\sum_{x,y}\left(a_{j}^{1}⊙a_{j}^{2}\right),$$

where ⊙ denotes element-wise multiplication, and *C* denotes the number of classes.

After applying GAP to the activation maps $M^{1}$ and $M^{2}$, we obtain the class scores $S^{1}$ and $S^{2}$ for both branches. To prevent one branch from becoming too dominant over the other branch during the competition process, we introduce an Unbalanced Score Penalty (BSP) to constrain $S^{1}$ and $S^{2}$, which is defined as follows:

(11) $$L_{bsp}=\frac{1}{C}\sum_{j=1}^{C}\left|S_{j}^{1}-S_{j}^{2}\right|.$$

This penalty $L_{bsp}$ is essentially the Mean Absolute Error (MAE) loss, which encourages balance between the two branches.

By taking the mean scores of the two branches, we obtain the final class scores $\bar{S}$. These scores are then normalized into a probability distribution $y\in R^{C}$ using the softmax function. The Focal Loss between the predicted probability *y* and the true label $\hat{t}$ is computed as follows:

(12) $$L_{fl}=-\sum_{j=1}^{C}\alpha_{j}{\left(1-y_{j}\right)}^{\gamma }{\hat{t}}_{j}\log\left(y_{j}\right),$$

where $\alpha_{j}$ is a class-balancing factor and is calculated from the class distribution of the training set, and γ is a focusing parameter that down-weights well-classified samples and is set to 2 in our implementation.

Integrating the above three losses, we obtain the total loss function for competitive fusion learning as follows:

(13) $$L_{total}=L_{fl}+\lambda \cdot L_{oap}+L_{bsp},$$

where $L_{fl}$ guides the model to learn acoustic features related to the class and dynamically scales cross-entropy to focus on hard examples, $L_{oap}$ enforces competitive learning between the two branches, and λ is a balancing weight. During training, λ gradually decreases from one to zero over epochs, resulting in stronger competition in early stages of training and weaker competition in later stages. $L_{bsp}$ guides the two branches to align and produce similar or identical class scores as the competition weakens.

## 4. Experiments and Results

### 4.1. Implementation Details

We implemented our method on an Ubuntu system equipped with a NVIDIA GTX 4090 (Santa Clara, CA, USA). During the data preprocessing stage, we used torchaudio [22] for audio processing. When extracting Mel spectrograms, the length of FFT window, the time interval between successive frames, and the number of Mel filters were, respectively, set to 0.16 s, 0.6 s, and 64. As a result, a one-minute long audio sample was converted into a tensor with dimensions of (1,64,1001). In all experiments, we adopted a leave-one-out cross-validation strategy [23]. Specifically, the data from one individual were selected as the test set while the data of the rest of the individuals were used for training, and the process was repeated for each individual. The batch size was set to 16. The learning rate was initialized as $10^{-4}$ and updated following a cosine annealing schedule [24]. The model was trained for 40 epochs.

### 4.2. Evaluation Metrics

We selected accuracy and F1-score as the evaluation metrics [25]. The F1-score balances both precision and recall. The definitions of these metrics are as follows:

- **Accuracy**: Accuracy is the proportion of correctly classified instances among all the test instances:
  (14) $$\mathrm{Accuracy}=\frac{TP+TN}{TP+TN+FP+FN},$$
  where TP is the number of true positives, TN is the number of true negatives, FP is the number of false positives, and FN is the number of false negatives.
- **F1-score**: The F1-score is the harmonic mean of precision and recall, providing a balanced measure of both:
  (15) $$F1-\mathrm{score}=2\times \frac{Precision\times Recall}{Precision+Recall},$$
  where Precision is defined as the proportion of correctly predicted positive instances among all of the predicted positive instances:
  (16) $$\mathrm{Precision}=\frac{TP}{TP+FP},$$
  and Recall is defined as the proportion of correctly predicted positive instances among all of the positive test instances:
  (17) $$\mathrm{Recall}=\frac{TP}{TP+FN}.$$

### 4.3. Results and Discussion

#### 4.3.1. Comparison with State-of-the-Art Methods

Table 2 provides a comparison of the proposed method with other state-of-the-art models, including BiLSTM [14], MobileNetV3 [26], ShuffleNetV2 [27], VGG16 [28], EfficientNetV2 [29], ResNet50 [19], ResNext50 [30], and HTS-AT [31], in terms of accuracy and F1-score. The results show that our method achieved an average accuracy (± standard deviation) of 92.98%±2.58% and an average F1-score (± standard deviation) of 92.99%±2.20%, outperforming other models in both classification accuracy and robustness.

**Figure 4:**
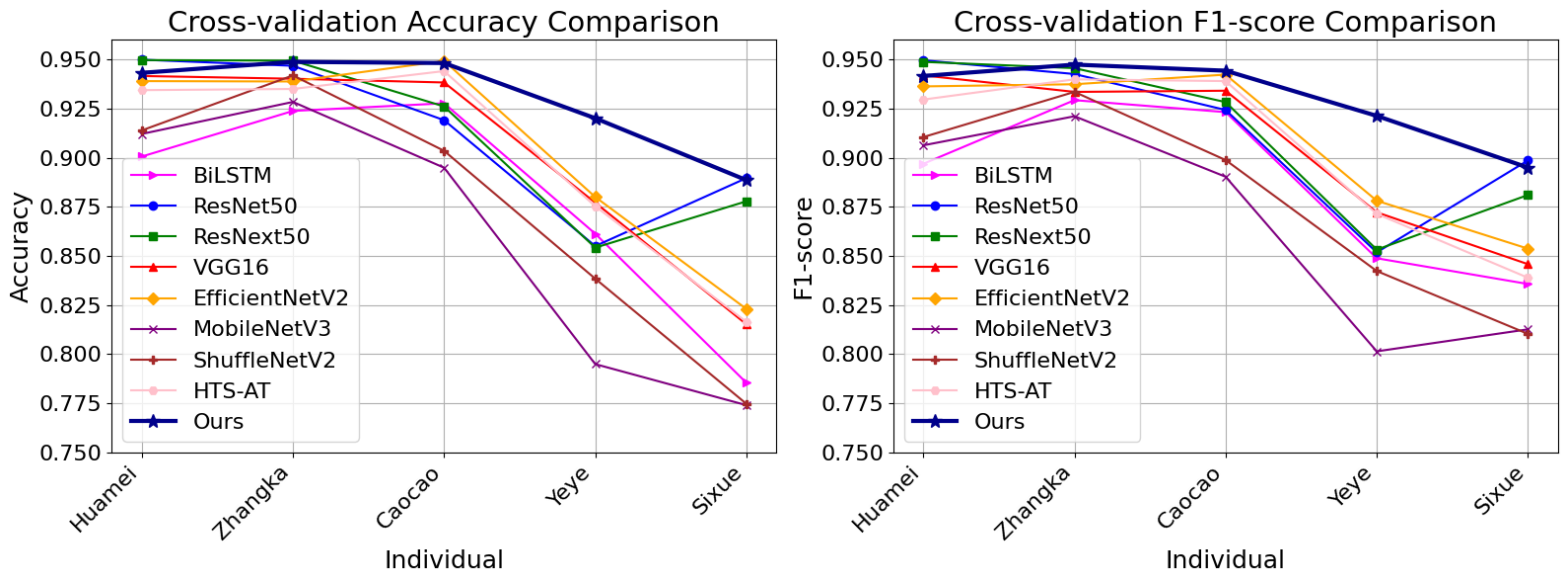
Accuracy and F1-score of different models for each individual in cross-validation.

Figure 4 shows the accuracy and F1-score of the methods under comparison across different panda individuals. From the results, we can see that our method is more stable than other methods in classifying new individual data and achieves the highest accuracy/F1-score for most individuals.

**Figure 5:**
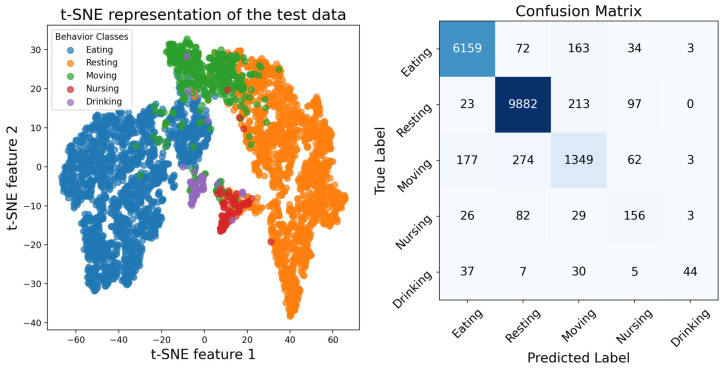
Visualization of classification performance of proposed method.

The t-distributed Stochastic Neighbor Embedding (t-SNE) plot provides a visual representation of the classification performance of some test data in a simplified two-dimensional feature space. For quantitative evaluation, the confusion matrix provides a detailed breakdown of the accuracy of the prediction between classes. As shown in Figure 5, most classes are correctly classified, such as eating and resting. However, there were some exceptions with a slightly lower proportion of correct classifications, such as nursing, which is often misclassified as resting. We infer that this misclassification occurs because giant panda mothers are usually in a relatively restful state while nursing their cubs, causing the model to misidentify them as resting. Additionally, the use of an acquisition device that stores data in MP3 compressed format may blur the cub’s vocalizations, making it difficult for the model to learn effective information. An important premise of using audio to identify the behavior of giant pandas is that giant pandas are solitary animals, and among the five behaviors, only nursing is a process of mother and baby together, which increases the difficulty of identification.

#### 4.3.2. Ablation Study

**Figure 6:**
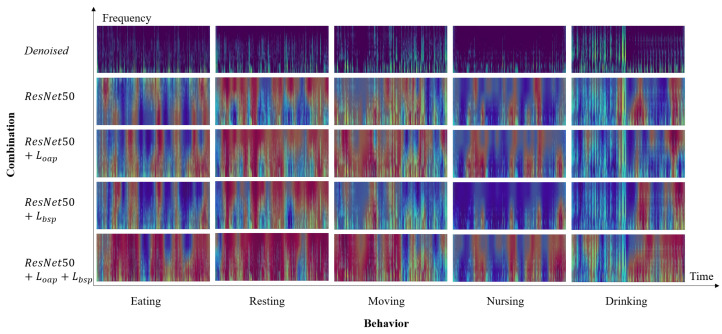
Visualization of class activation maps for selected samples.

To evaluate the effectiveness of denoising, as well as the impact of $L_{oap}$ and $L_{bsp}$, we conducted ablation experiments in this section. The results of the ablation study are shown in Table 3. A comparison of the first two rows indicates that denoising influences the recognition performance, highlighting the considerable impact of noise on behavior classification. As the recorder is tied to the collar, denoising mainly reduces the friction sound, wind, and other noises of the collar, which can avoid the noise masking the behavior features we need. Furthermore, a comparison of the last four rows demonstrates that the combination of $L_{oap}$ and $L_{bsp}$ enables the two branches of the model to learn more basic features or combinations of features (see Figure 6) in competition and balance and improves the feature representation ability of the model, and they are all indispensable.

Additionally, Figure 6 presents the denoised Mel spectrograms and CAMs for some samples. It is evident that by employing competitive fusion learning, the model is able to capture more diverse audio features such as chewing, rhythmic swallowing sounds, and vocalization bursts. For example, in the sample of drinking in Figure 6, the beginning of the entire audio clip is a giant panda doing other activities, and the latter part is drinking water. There is an obvious rhythmic gurgling sound in the Mel spectrogram. After using the competitive fusion learning method that includes $L_{oap}$ and $L_{bsp}$, the model is able to learn to express behavior features more accurately, which correspond to the time and frequency ranges of more basic acoustic events on the Mel spectrogram.

**Figure 7:**
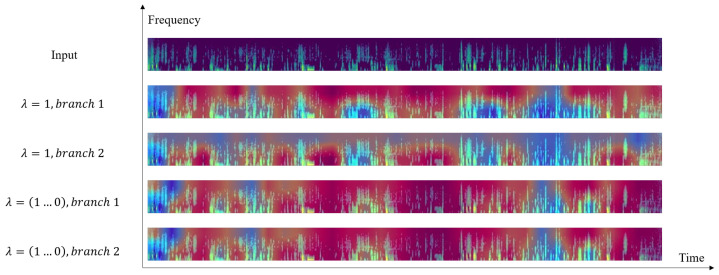
Visualization of each branch’s class activation maps for selected samples.

To evaluate the effectiveness of setting the dynamic parameter λ, we compare the CAMs generated by the dual-branch under two configurations: (1) a fixed λ=1 and (2) a linearly decaying λ, as shown in Figure 7. When λ=1, the two branches produce different outputs, necessitating collaborative inference from both branches. In contrast, when λ decays dynamically, the branches converge to identical outputs. Therefore, we can obtain the same result using only a single branch while reducing computational costs.

#### 4.3.3. Different Backbones

In order to test the universality and effectiveness of our proposed framework, we conducted experiments on different CNN-based backbones. Table 4 presents the experimental results of competitive fusion learning on different backbones, including ResNet50 [19], VGG16 [28], MobileNetV1 [32], MobileNetV2 [33], MobileNetV3 [26], ShuffleNetV2 [27], and EfficientNetB0 [34]. The results show that the proposed method achieves a significant improvement in accuracy and F1-score by 1–2% on average and a reduction in the standard deviation by 1–2% on these backbones, further validating the effectiveness and broad applicability of our method. Notably, the last five models are lightweight architectures, which have significantly fewer parameters and lower computational costs compared to the others. This suggests that our method offers a promising solution for enhancing algorithm performance and enabling deployment on mobile terminals or edge devices.

## 5. Conclusions

In order to explore the potential of audio-based automatic recognition of panda behaviors, we have collected an audio dataset containing 18,930 samples of five behaviors from five individual pandas. In this study, we propose a novel audio-based GPBR method armed with competitive fusion learning. Our method is capable of extracting complex acoustic features, which significantly improves recognition performance and robustness without extra computational burden during the inference stage.

We conducted extensive experiments, and the results demonstrate that our method outperforms other deep learning approaches. These results also validate the feasibility of audio-based GPBR. Furthermore, we applied competitive fusion learning to various CNN backbones, achieving significant performance improvements, particularly with lightweight models. This opens up new possibilities for enhancing algorithm performance and deploying the system on mobile devices. Ultimately, this approach not only facilitates panda monitoring but can also be extended to studies of other species, providing a powerful tool for global wildlife conservation efforts.

Despite these significant advances, audio-based GPBR still faces substantial challenges, such as the scarcity of labeled data, noise interference, and behavioral differences between adult and juvenile individuals. In future work, we plan to further expand the dataset and refine the method to address these challenges for better performance of audio-based GPBR.

## Figures and Tables

**Table 1:** The number of samples for each behavior of each individual.

Individual Name	Behavior					Total
	Eating	Resting	Moving	Nursing	Drinking	
Huamei	1688	1622	474	51	20	3855
Zhangka	631	2662	109	75	2	3479
Caocao	1451	2028	290	48	26	3843
Yeye	1706	1530	415	87	55	3793
Sixue	955	2373	577	35	20	3960
Total	6431	10,215	1865	296	123	18,930

**Table 2:** Comparison of the proposed method with other deep learning methods.

Methods	Accuracy (%)	F1-Score (%)
BiLSTM	87.98 ± 5.90	88.68 ± 4.27
MobileNetV3	86.09 ± 7.11	86.63 ± 5.54
ShuffleNetV2	87.45 ± 6.75	87.91 ± 5.10
VGG16	90.25 ± 5.59	90.55 ± 4.36
EfficientNetV2	90.60 ± 5.39	90.96 ± 4.08
ResNet50	91.21 ± 4.02	91.34 ± 3.97
ResNext50	91.14 ± 4.34	91.14 ± 4.23
HTS-AT	90.10 ± 5.47	90.38 ± 4.60
**Proposed**	**92.98 ± 2.58**	**92.99 ± 2.20**

The bold entries are meant to show the best performance.

**Table 3:** The results of ablation studies with our method.

Denoised	$L_{\mathrm{oap}}$	$L_{\mathrm{bsp}}$	Accuracy (%)	F1-Score (%)
—	—	—	88.73 ± 7.72	88.73 ± 7.35
✓	—	—	91.21 ± 4.02	91.34 ± 3.97
✓	✓	—	91.11 ± 3.35	91.35 ± 2.80
✓	—	✓	91.54 ± 3.60	91.50 ± 3.34
✓	✓	✓	**92.98 ± 2.58**	**92.99 ± 2.20**

The bold entries are meant to show the best performance.

**Table 4:** The results of different backbones with competitive fusion learning.

Backbone	Baseline		Competitive Fusion Learning		Params (M)	FLOPS (M)
	Accuracy (%)	F1-Score (%)	Accuracy (%)	F1-Score (%)		
ResNet50	91.21 ± 4.02	91.34 ± 3.97	92.98 ± 2.58	92.99 ± 2.20	23.51	5221.14
VGG16	90.25 ± 5.59	90.55 ± 4.36	91.57 ± 2.78	90.27 ± 2.77	134.28	22,477.86
MobileNetV1	88.53 ± 6.49	88.97 ± 5.11	89.04 ± 5.46	89.02 ± 5.05	3.19	721.62
MobileNetV2	87.01 ± 7.26	87.65 ± 5.58	88.57 ± 6.21	88.83 ± 5.18	2.20	376.98
MobileNetV3	86.09 ± 7.11	86.63 ± 5.54	87.21 ± 6.65	87.99 ± 5.40	1.51	66.73
ShuffleNetV2	87.45 ± 6.74	87.91 ± 5.10	88.50 ± 5.20	88.62 ± 4.42	1.26	188.94
EfficientNetB0	89.02 ± 5.02	88.87 ± 4.20	89.23 ± 5.64	89.71 ± 4.50	3.97	487.10

## Data Availability

All data generated or presented in this study are available upon request from the corresponding author. Furthermore, the models and code used during this study cannot be shared at this time as the data also form part of an ongoing study.
